# Prevalence of Cholesteatoma in School Children of Nepal

**DOI:** 10.1055/s-0045-1811514

**Published:** 2025-10-16

**Authors:** Milan Maharjan, Samira Rajbhandari, Shristi Subba, Anjani Gupta

**Affiliations:** 1 Department of Ear, Nose, and Throat, Ear Care Nepal, Star Hospital, Sanepa Height, Lalitpur, Nepal

**Keywords:** cholesteatoma, prevalence, children, hearing loss, Nepal

## Abstract

**Introduction:**

Cholesteatoma remains a serious condition that poses a challenge to otolaryngologists around the world. It is found to be more aggressive in the pediatric group than in adults. If left untreated, the disease can be dangerous due to its ability to expand and destroy bones, leading to complications such as hearing loss, vestibular dysfunction, facial paralysis, and intracranial infections.

**Objective:**

To find out the prevalence of cholesteatoma in the school-aged children of Nepal.

**Methods:**

This is a retrospective study conducted within a span of 10 years from 2014 to 2024 in which all medical records of the school-based ear screening programs were studied. Data including personal details, brief history, and ear and audiometry findings were recorded. All suspected cases of cholesteatoma were reexamined under microscope at our institute, and only those with confirmed diagnosis of chronic otitis media (COM) with cholesteatoma were included in the study.

**Results:**

Out of the total of 143,544 children screened, COM with cholesteatoma was diagnosed in 0.20% (n = 282), and COM in total in 7.56% (n = 10,853). Hearing loss was seen in 273 (96.81%) of the 282 cases, with conductive hearing loss being the most common type of hearing loss.

**Conclusion:**

There is a higher prevalence of cholesteatoma in Nepalese children. Early diagnosis with proper management helps reduce the chances of life-threatening complications. Thus, having regular screening programs in school children will help in reducing morbidity.

## Introduction

Cholesteatoma is considered an intriguing and complex topic in otology, which has encouraged much research and debate worldwide. 1 This disease is defined as a mass formed by proliferative keratinizing squamous epithelium and subepithelial connective tissue with progressive accumulation of keratin debris with or without surrounding inflammatory reaction in the tympanic cavity and/or mastoid. 2 Cholesteatomas can be potentially dangerous due to their ability to expand and destroy bone, leading to complications such as hearing loss, vestibular dysfunction, facial paralysis, and intracranial infection. 3

It is estimated that over 20 million people worldwide are affected by chronic otitis media (COM). Of these, one fourth (about 5 million) have cholesteatomas. 1 The annual incidence of this condition ranges from 9 to 12.6 cases per 100,000 adults, and from 3 to 15 cases per 100,000 children. 4 5 The disease is known to be more extensive in children compared to adults, with higher rates of morbidity and recurrence being documented. 6 7 8 A well-pneumatized mastoid in children allows space for spread of disease, compared to a more sclerotic mastoid in adults. Also, immature anatomy and dysfunction of the Eustachian tube leads to more frequent middle ear infections. Small anatomy, a difficult examination, and more aggressive disease make these pathologies difficult to diagnose and treat. 8 9 10 11 12 Timely detection with prompt and effective treatment greatly reduces the frequency of complications and worse prognosis. 13

This shows the importance of undertaking a study like this in children. Cholesteatoma studies have not been done in Nepal, so its true prevalence is unknown in our community. Therefore, the main objective of our study is to find out the prevalence of cholesteatoma in Nepalese children.

## Methods

This is a retrospective study. Medical records and pro forma of school ear screening programs conducted by our institute over a period of ten years from 2014 to 2024 were analyzed. All children belonging to grade 1 through 10, aged 5 to 16 years were included in the study. Pro forma containing personal details such as age, gender, grade and a brief history were collected.

Ear, Nose & Throat surgeons did all the ear examinations by using a Heine Mini 3000 Otoscope. Detail otoscopic findings such as presence of cholesteatoma and perforations with its sites were recorded. Diagnosed cases of COM with cholesteatoma were referred to a tertiary level hospital setting for microscopic examination for final confirmation of cholesteatoma. Active squamous COM synonymously called cholesteatoma was diagnosed when there was a retraction of the pars flaccida or tensa with retained squamous epithelial debris associated with inflammation and the production of pus. Only cases of COM with cholesteatoma confirmed by microscopic evaluation were included in the study. Cases without active cholesteatoma or children who did not come for microscopic evaluation were excluded from the study. Other types of otitis media such as COM mucosal type, retraction pockets, tympanosclerosis, adhesive otitis media and other pathologies of the middle ear were also excluded from the study. Due to financial constraints, temporal bone computed tomography (CT) scan was not done.

Detailed findings of the microscopic examination of all the children were documented. Pure tone audiometry was conducted by an audio-technician using an Arphi Proton DX3 pure tone audiometer. Pure tone average for air and bone conduction was measured across all frequencies and average hearing threshold was calculated at 500, 1,000, 2,000, and 4,000 Hz. Hearing loss (HL) was defined as a pure tone average of four frequencies greater than 25dB HL. Hearing loss was graded according to the American Speech-Language Hearing Association (ASHA) into 5 grades: 1) Mild: 26–40 dB HL, 2) Moderate: 41–55 dB HL, 3) Moderately severe: 56–70 dB HL, 4) Severe: 71–90 dB HL, and 5) Profound: > 91 dB HL. 14

The ethical clearance to conduct the study was approved by the Nepal Health Research Council (NHRC). Data entry and analysis was done by using Microsoft Excel 2013 (Microsoft Corp.) and the SPSS Statistics for Windows (IBM Corp.), version 25.0 software.

## Results

A total of 143,544 children from grade 1 to 10 were screened over a period of 10 years from 2014 to 2024. COM was seen in 7.56% (n = 10,853) of the children. Out of the total screened, COM with cholesteatoma was diagnosed in 0.20% (n = 282). Among the cholesteatoma cases, 46.81% (n = 132) were girls and 53.19% (n = 150) were boys.

Children between the ages of 5 to 16 years were included. The highest incidence was seen in the 11-to-16-year-old group, with 78.37% (n = 221), followed by the 5-to-10 years group 21.63% (n = 61). Regarding laterality of the disease, 31.21% (n = 88) were present in right ear, 58.86% (n = 166) in left, and 9.93% (n = 28) in both. The demographic profile of children with cholesteatoma is shown in Table 1 .

**Table 1:** Demographic information of children with cholesteatoma

Variables	Category		Number	%
Age (years)	5–10		61	21.63
	11–16		221	78.37
Gender	Male		150	53.19
	Female		132	46.81
Laterality	Right		88	31.21
	Left		166	58.86
	Both		28	9.93
Ethnicity	Brahmin		53	18.79
	Chhetri		50	17.73
	Janajati		127	45.04
	Dalit		28	9.93
	Madhesi		23	8.16
	Others		1	0.35
Hearing loss	CHL	Mild	183	72.05
		Moderate	52	20.47
		Moderately severe	19	7.48
	SNHL		0	0
	Mixed		19	6.74
	Normal		9	3.19

**Abbreviations:** CHL, conductive hearing loss; SNHL, sensorineural hearing loss.

Hearing loss was seen in 273 (96.81%) among the 282 cases of COM with cholesteatoma. Conductive hearing loss (CHL) was the most prevalent type, followed by mixed type. The details of hearing loss across different age groups are presented in Table 2 .

**Table 2:** Type of hearing loss according to age group

Age distribution (years)	Type and degree of hearing loss n (%)		
**5–10**	CHL (96.72%)	Mild	43 (72.88%)
		Moderate	13 (22.03%)
		Moderately severe	3 (5.09%)
	Mixed hearing loss	Moderate	2 (3.28%)
**11–16**	CHL (88.24%)	Mild	140 (71.79%)
		Moderate	39 (20.00%)
		Moderately severe	16 (8.21%)
	Mixed hearing loss (7.69%)	Moderate	10 (58.82%)
		Moderately severe	7 (41.18%)
	Normal		9 (4.07%)

**Abbreviation:** CHL, conductive hearing loss.

## Discussion

Cholesteatoma continues to be a challenge for otolaryngologists around the world, especially in the pediatric population, due to its aggressive nature and increased chances of life threatening complications. 15 16 17 18 There is a significant prevalence of cholesteatoma and its complications not only in developing countries like ours but also in those with advanced healthcare facilities where there is easy access to healthcare and specialists. 15 So, this study has tried to show the prevalence of the disease in children, thereby showcasing its burden in our community.

The true incidence of pediatric cholesteatoma is not known, and epidemiological studies are scarce. 19 There have been no studies to date regarding cholesteatoma prevalence in Nepal, which makes it difficult to compare our results with other national studies. However, similar international papers are available for comparison. The prevalence of cholesteatoma among the 143,544 children aged 5 to 16 years of age was 0.20% in our study. This is higher than a study conducted by Dorney et al. in the United States, which showed 0.09% cholesteatoma cases in patients 18-years-old or younger. 20

Similarly, a nationwide study in Denmark described the incidence rate of surgically treated middle ear cholesteatoma in 3,874 Danish children (0–15 years), from 1977 to 2010, to be 0.08 to 0.15%. 21 Tos et al. showed an incidence of 0.03% among surgically treated children. 4 An epidemiological study done in Fukuoka, Japan, in 2008, showed an incidence of cholesteatoma of 0.06 to 0.1%. 22 Khalid-Raja et al. noted the mastoid surgery rate was similar to the incidence rate of cholesteatoma, since it is mostly managed surgically, finding an intervention rate of 0.11% in their study population. 23

The higher prevalence in our study may be due to the population belonging to government and Buddhist monastic schools, where most children are from low socioeconomic background. So, risk factors including poor hygiene, malnutrition, overcrowding, frequent upper respiratory tract infections, low access to medical care, and exposure to passive smoking are more common. 24 25 26

The incidence of cholesteatoma in COM cases was 2.59% in our study. This is lower compared to a study done by Mathema et al., 27 which had an incidence of 8.11%. This high prevalence could be because it was conducted in a specialized hospital for ear problems. Kamal et al. 28 showed a 6.7% rate of cholesteatoma cases in the 203 samples examined. Such high incidence may be because the study was done in the slum dwellers of Dhaka city. Also, both the studies had a small population.

Cholesteatoma was found to be more common in males than in females, similar to the findings of other studies. 8 29 30 Regarding laterality of the disease, left ear was affected more than the right. Of the cholesteatoma cases, 9.93% had bilateral disease, which is higher than the finding by Kemppainen et al. (4.4%). 31

The disease was more prevalent in older children of the 11-to-16 years age group than in younger ones aged 5 to 10 years. A retrospective study done by Khdim et al. in 100 cholesteatoma patients also showed a higher incidence in 10 to 20-year-old age group. 32 Also, an Indian study by Nagaraj et al. in the pediatric population had more proportion in 13 to 16-year-olds. 33

Among the different ethnic groups in Nepal, higher number of cholesteatoma cases were seen in Janajatis (45.04%), followed by Brahmins (18.79%) and Chhetris (17.73%). Janajati is the term used to identify the ethnic indigenous people of Nepal. They comprise the majority of the population in our country. According to the 2021 national census, 27.38% of the total population identify as Janajatis. They are considered an underprivileged population group, with lower human development index, per capita income, and education. 34 35 This may explain the higher incidence of the disease in this ethnic group.

Hearing loss was seen in 96.81% of the cholesteatoma cases. This high incidence may be due to the ossicular involvement by the disease leading to decrease in hearing threshold. 36 The most common type of hearing loss was conductive, with a 90.07% incidence. A study done in 2018 in Karnataka, India, showed conductive hearing loss in 83% of the population, which is a high incidence and similar to our findings. 37

The most predominant grade of CHL in this study was mild, with a 72.05% incidence followed by moderate and moderately severe. Similar findings were seen in studies done by Stern et al. and Visvanathan et al., where the mild degree of hearing loss was more common. 36 38

Additionally, 3.19% of our patients had a normal audition. This can be due to the cholesteatoma sac artificially maintaining the continuity of the ossicular chain in the inner ear, ensuring the transmission of sound vibrations. 3

This study has several limitations. Firstly, the focus on government schools means the findings may not reflect the accurate prevalence of the disease among all school-aged children in Nepal, as private schools were excluded. In Nepal, many of the children attend government-run schools due to financial constraints, which indirectly represents the true pediatric population of Nepal. Secondly, due to limited resources, CT scans of the temporal bone were not done to confirm the diagnosis. Regardless of the limitations, this study is the only one done in Nepal involving such a large study population.

## Conclusion

This is the only large population study done for cholesteatoma in children in Nepal. The results show a higher prevalence prevalence than in other parts of the world, indicating the need for further research and attention. It also demonstrates a higher prevalence in underprivileged ethnicities and demographics in Nepal, indicating a need for interventions focused on healthcare equity. Early diagnosis with proper management helps to diminish the chances of developing life-threatening complications. Thus, having regular screening programs in school children will help to reduce morbidity.

## References

[1] de Aquino J EAP Cruz Filho N A de Aquino J NP Epidemiology of middle ear and mastoid cholesteatomas: study of 1146 cases Braz J Otorhinolaryngol 2011 77 03 341 347 10.1590/s1808-86942011000300012 21739009 PMC9443752

[2] Yung M Tono T Olszewska E Yamamoto Y Sudhoff H Sakagami M EAONO/JOS joint consensus statements on the definitions, classification and staging of middle ear cholesteatoma J Int Adv Otol 2017 13 01 1 8 10.5152/iao.2017.3363 28059056

[3] Browning G G Weir J Kelly G Swan I RC Chronic Otitis Media 8th ed. Boca Raton CRC Press 2018 977 1014

[4] Tos M Incidence, etiology and pathogenesis of cholesteatoma in children Adv Otorhinolaryngol 1988 40 110 117 10.1159/000415679 3389223

[5] Kuo C L Shiao A S Yung M Sakagami M Sudhoff H Wang C H Updates and knowledge gaps in cholesteatoma research Biomed Res Int 2015 2015 854024 10.1155/2015/854024 25866816 PMC4381684

[6] Glasscock M E III Dickins J R Wiet R Cholesteatoma in children Laryngoscope 1981 91 10 1743 1753 10.1288/00005537-198110000-00021 7289705

[7] Palva A Karma P Kärjä J Cholesteatoma in children Arch Otolaryngol 1977 103 02 74 77 10.1001/archotol.1977.00780190054003 836233

[8] Lynrah Z A Bakshi J Panda N K Khandelwal N K Aggressiveness of pediatric cholesteatoma. Do we have an evidence? Indian J Otolaryngol Head Neck Surg 2013 65 03 264 268 10.1007/s12070-012-0548-z 24427579 PMC3696163

[9] Hildmann H Sudhoff H Cholesteatoma in children Int J Pediatr Otorhinolaryngol 1999 49 01 S81 S86 10.1016/s0165-5876(99)00138-x 10577781

[10] Preciado D A Biology of cholesteatoma: special considerations in pediatric patients Int J Pediatr Otorhinolaryngol 2012 76 03 319 321 10.1016/j.ijporl.2011.12.014 22264818

[11] Dornhoffer J L Friedman A B Gluth M B Management of acquired cholesteatoma in the pediatric population Curr Opin Otolaryngol Head Neck Surg 2013 21 05 440 445 10.1097/MOO.0b013e32836464bd 23872727

[12] Luu K Chi D Kiyosaki K K Chang K W Updates in pediatric cholesteatoma: Minimizing intervention while maximizing outcomes Otolaryngol Clin North Am 2019 52 05 813 823 10.1016/j.otc.2019.05.003 31280890

[13] Baig M M Ajmal M A Saeed I Fatima S Prevalence of cholesteatoma and its complications in patients of chronic suppurative otitis media J Rawalpindi Med Coll 2011 15 01 16 17

[14] Clark J G Uses and abuses of hearing loss classification ASHA 1981 23 07 493 500 7052898

[15] Rutkowska J Özgirgin N Olszewska E Cholesteatoma definition and classification: A literature review J Int Adv Otol 2017 13 02 266 271 10.5152/iao.2017.3411 28274903

[16] Miller K M Liu Y C Weinstein J E Cohen M S Chi D H Anne S Outcomes in pediatric cholesteatoma Otolaryngol Head Neck Surg 2025 172 01 299 306 10.1002/ohn.1025 39441616 PMC11697529

[17] Miller K A Fina M Lee D J Principles of pediatric endoscopic ear surgery Otolaryngol Clin North Am 2019 52 05 825 845 10.1016/j.otc.2019.06.001 31353138

[18] Lima A F Moreira F C Menezes A S Costa I E Azevedo C Breda M S Dias L Is pediatric cholesteatoma more aggressive in children than in adults? A comparative study using the EAONO/JOS classification Int J Pediatr Otorhinolaryngol 2020 138 110170 10.1016/j.ijporl.2020.110170 32705986

[19] Ryan P J Patel N P Endoscopic management of pediatric cholesteatoma J Otol 2020 15 01 17 26 10.1016/j.joto.2018.11.009 32110236 PMC7033597

[20] Dorney I Otteson T Kaelber D C Middle ear cholesteatoma prevalence in over 3,600 children with Turner Syndrome Int J Pediatr Otorhinolaryngol 2022 161 111289 10.1016/j.ijporl.2022.111289 35987131

[21] Djurhuus B D Skytthe A Christensen K Faber C E Cholesteatoma in Danish children - a national study of changes in the incidence rate over 34 years Int J Pediatr Otorhinolaryngol 2015 79 02 127 130 10.1016/j.ijporl.2014.11.020 25496822

[22] Shibata S Murakami K Umeno Y Komune S Epidemiological study of cholesteatoma in Fukuoka City J Laryngol Otol 2015 129 02 S6 S11 10.1017/S002221511400231X 25706164

[23] Khalid-Raja M Tikka T Coulson C Cholesteatoma: a disease of the poor (socially deprived)? Eur Arch Otorhinolaryngol 2015 272 10 2799 2805 10.1007/s00405-014-3285-y 25231708

[24] Srikanth S Isaac R Rebekah G Rupa V Knowledge, attitudes and practices with respect to risk factors for otitis media in a rural South Indian community Int J Pediatr Otorhinolaryngol 2009 73 10 1394 1398 10.1016/j.ijporl.2009.06.024 19640593

[25] Mukara K B Lilford R J Tucci D L Waiswa P Prevalence of middle ear infections and associated risk factors in children under 5 years in Gasabo district of Kigali City, Rwanda Int J Pediatr 2017 2017 4.280583E6 10.1155/2017/4280583 PMC5733628 29348761

[26] Tapia M Schmidt T Prevalence of middle ear disease in Chilean natives and the impact of development over 14 years Braz J Otorhinolaryngol 2021 87 03 283 289 10.1016/j.bjorl.2019.09.004 31753782 PMC9422723

[27] Mathema L Adhikari A Poudyal P Chalise G B Chaudhary P Khatri B Chronic Otitis Media among patients visiting community-based static outreach clinics JNMA J Nepal Med Assoc 2023 61 268 923 926 10.31729/jnma.8369 38289754 PMC10792720

[28] Kamal N Joarder A H Chowdhury A A Khan A W Prevalence of chronic suppurative otitis media among the children living in two selected slums of Dhaka City Bangladesh Med Res Counc Bull 2004 30 03 95 104 16240980

[29] Diom E S Cisse Z Tall A Ndiaye M Pegbessou E Ndiaye I C Management of acquired cholesteatoma in children: a 15 year review in ENT service of CHNU de FANN Dakar Int J Pediatr Otorhinolaryngol 2013 77 12 1998 2003 10.1016/j.ijporl.2013.09.021 24148865

[30] Olszewska E Wagner M Bernal-Sprekelsen M Ebmeyer J Dazert S Hildmann H Sudhoff H Etiopathogenesis of cholesteatoma Eur Arch Otorhinolaryngol 2004 261 01 6 24 10.1007/s00405-003-0623-x 12835944

[31] Kemppainen H O Puhakka H J Laippala P J Sipilä M M Manninen M P Karma P H Epidemiology and aetiology of middle ear cholesteatoma Acta Otolaryngol 1999 119 05 568 572 10.1080/00016489950180801 10478597

[32] Khdim M Douimi L Choukry K Oukessou Y Rouadi S Abada R Hearing Loss in Cholesteatoma Am J Otolaryngol Head Neck Surg 2020 3 08 1115

[33] Nagaraj K Ravishankar V Srinivas K Babu S Rathod J BS Reddy D S A prospective study of cholesteatoma in paediatric age group and its management J. Evid. Based Med. Healthc 2016 3 11 321 324 10.18410/jebmh/2016/77

[34] National Statistics Office National Population and Housing Census 2021 2021. Available at:https://censusnepal.cbs.gov.np/results/downloads/caste-ethnicity?type=report

[35] Gautam D Sharma G R Social Inclusion and Underdevelopment of Nepalese ‘Adivasi Janajatis’ Administration and Management Review 2011 23 1 9

[36] Stern S J Fazekas-May M Cholesteatoma in the pediatric population: prognostic indicators for surgical decision making Laryngoscope 1992 102(12 Pt 1):1349 1352 10.1288/00005537-199212000-00007 1453840

[37] Shwetha N Chronic otitis media with cholesteatoma: clinical presentation and surgical management Int J Otorhinolaryngol Head Neck Surg 2018 4 05 1212 1219 10.18203/issn.2454-5929.ijohns20183454

[38] Visvanathan V Kubba H Morrissey M SC Cholesteatoma surgery in children: 10-year retrospective review J Laryngol Otol 2012 126 05 450 453 10.1017/S0022215111003483 22310087

